# The Impact of a Large Bolus Dose of l-leucine and l-isoleucine on Enteroendocrine and Pancreatic Hormones, and Glycemia in Healthy, Inactive Adults

**DOI:** 10.3390/nu11112650

**Published:** 2019-11-04

**Authors:** Daniel E. Newmire, Eric Rivas, Sarah E. Deemer, Darryn S. Willoughby, Victor Ben-Ezra

**Affiliations:** 1 Exercise Physiology and Biochemistry Lab, Department of Kinesiology, Texas A&M University-Corpus Christi, Corpus Christi, TX 78414, USA; 2 Exercise and Thermal Integrative Physiology Laboratory, Department of Kinesiology and Sports Management, Texas Tech University, Lubbock, TX 79409, USA; eric.rivas@ttu.edu; 3 Nutrition Obesity Research Center, University of Alabama-Birmingham, Birmingham, AL 35233, USA; sdeemer@uab.edu; 4 Exercise and Biochemical Nutrition Laboratory, Department of Health, Human Performance, Recreation, Baylor University, Waco, TX 76707, USA; Darryn_Willoughby@baylor.edu; 5 Exercise Physiology and Biochemistry Lab, Department of Kinesiology, Texas Woman’s University, Denton, TX 76204, USA; VBenEzra@twu.edu

**Keywords:** L-isoleucine, L-leucine, glucagon-like peptide 1, glucose-dependent insulinotropic peptide, glycemia

## Abstract

Background: The ingestion of whey protein and amino acids with carbohydrate (CHO) enhances the release of glucagon-like peptide-1 (GLP-1) and glucose-dependent-insulinotropic peptide (GIP) that promote insulin secretion. It is unknown if L-isoleucine (Ile) and L-leucine (Leu) have this same effect. The purpose of this study was to examine how Ile and Leu influence both GLP-1 and GIP, subsequent pancreatic hormones, and glycemia in healthy, inactive adults. Methods: Twelve adults (6F/6M; age 27.4 ± 2 years; BMI 26.3 ± 2 kg/m^2^; lean body mass 53.2 ± 5 kg; body fat 34.1 ± 3%) completed four conditions in a randomized, cross-over fashion. Treatments standardized (0.3 g/kg·LBM^−1^) (1) Leu, (2) Ile, (3) Equal (1:1 g) of Leu + Ile, and (4) placebo (Pla, 3.5 g inert stevia) ingested 30 min prior to an oral glucose tolerance test (OGTT). Samples of plasma glucose, insulin, glucagon, GIP_Total_, and GLP-1_Active_ were assessed. Results: A treatment (*p* = 0.01) effect comparing Ile vs. Leu (*p* = 0.02) in GIP_Total_. Area under the curve showed an increase in GIP_Total_ from Ile compared to Leu and Pla (*p* = 0.03). No effect was found on GLP-1. The ingestion of Ile prior to CHO augmented GIP concentration greater than Leu or Pla. No correlation was found between GIP, insulin, and glucose between conditions. Conclusions: Ile impacts GIP concentration, which did not relate to either insulin or glucose concentrations. Neither Ile, nor Leu seem to have an effect on hyperglycemia ingested prior to a CHO drink.

## 1. Introduction

More than 100 million U.S. adults are now living with diabetes or prediabetes, with 90% to 95% diagnosed with type 2 diabetes (T2D) according to the Centers for Disease Control and Prevention (CDC), with an economic burden of up to ~$245 billion [1,2,3]. Therapies other than pharmacological assistance include long-term weight control, which normally involves combinations of diet, exercise, and behavior modification that improve glucose control, lipids, blood pressure, and body composition profiles [4].

Previous research has found protein intake suppresses glycemic responses [5,6,7,8,9,10,11]. Furthermore, whey protein consumed prior to or with a meal in normal or T2D subjects has shown a positive impact on metabolic responses [12,13,14,15]. It has been suggested that the large amount of branched-chain amino acids (BCAAs) found in whey protein may promote hyperinsulinemia by inducing an insulinogenic effect [16,17,18] which influences the skeletal muscle uptake of glucose by potentially directly and indirectly stimulating insulin cell signaling pathways [19,20] and possibly through the incretin effect [21]. However, the relationship between amino acid ingestion, the incretin effect, and glycemic control is not well understood in humans. The incretin hormones glucagon-like peptide 1 (GLP-1) and glucose-dependent insulinotropic polypeptide (GIP) are released from the small and large intestines and respond mainly to glucose or lipid ingestion, which enhances glucose-stimulated insulin secretion [22,23]. GLP-1 is synthesized and secreted by L-cells found in the distal small intestines (ileum) and colon. Roughly, 80% is secreted as GLP-1 (7–36), which is the primary form of GLP-1 [24]. GLP-1 (7–37) is the degraded form of GLP-1 through the enzymatic action of dipeptidyl peptidase IV (DPP-IV). GLP-1 also influences pancreatic β-cell sensitivity to glucose and thus enhances glucose-dependent insulin secretion while concurrently improving the sensitivity to glucose of the α-cells under hyperglycemic conditions [25].

The other incretin hormone, GIP, also enhances the sensitivity of the α-cells to glucose under hypoglycemic conditions and stimulates the enhancement of glucagon counter-regulation to maintain glycemic homeostasis [26,27]. GIP is synthesized and released by enterocyte K-cells present in the proximal region both the duodenum and jejunum that responds mainly to glucose or lipid ingestion, which enhances glucose-stimulated insulin secretion. It has been proposed that the incretin effect is responsible for driving ~50% to 70% of postprandial insulin response after carbohydrate (CHO) ingestion, in healthy individuals [22] while impaired in T2Ds [28].

The compensatory hyperinsulinemia in response to CHO ingestion and hyperglycemia may be impaired in long-term diabetics. It has been suggested that β-cells synthesis and secretion capacity of insulin has been compromised. However, previous literature has suggested that this capacity may be nutritionally augmented with the co-ingestion of CHO and amino acids [29]. More notably, BCAA’s L-leucine (Leu) and L-isoleucine (Ile) have shown to have a positive effect on glycemia and have gained interest as a potential nutritional therapy [19].

The actions of Leu [30,31,32] and Ile [20,33,34,35] have shown to positively affect hyperglycemia through differing mechanisms depending in both human and rodent models. Recently, Lindgren et al. (2015) investigated the effect of an orally ingested amino acid mixture on glycemic responses in humans and found a positive effect on GIP response [21]. Additionally, a group investigated the effect of either Leu and Ile on glycemic and incretin responses and found a reduction in blood glucose where Leu stimulated an increase in insulin independent of incretin response and Ile reduced blood glucose independent of both the incretins and insulin [36,37]. However, it is largely unknown if this mediated glycemic action was influenced by the mixture of amino acids, an individual amino acid, or a direct or indirect amino acid-induced hormone response. Additionally, if Ile and Leu do indeed influence glucose uptake as suggested by previous literature, it is unknown how they would affect glycemia prior to a CHO drink. Therefore, this study design was constructed to determine how a standardized, large oral bolus of Leu and Ile ingested prior to CHO drink affect GIP and GLP-1 responses, their interrelated pancreatic hormones glucagon and insulin, and glycemia.

## 2. Materials and Methods

### 2.1. Participants and Ethics Approval

Twelve-healthy, self-reported inactive adults, which was defined as less than the ACSM recommended 30 min·day^−1^ or 150 min·week^−1^ of physical activity [38] (mean ± SEM; 6 M/F; age 27.4 ± 2.0 y; lean body mass (LBM) 48.6 ± 4.67 kg; body fat 34.1 ± 2.96%) volunteered to participate in this study (Table 1). The study design, purpose, and potential risks associated with the study were explained in detail to participants before obtaining written informed consent. Exclusion criteria were self-reporting a physically active lifestyle [38], metabolic disorders, current participation in other study trials, blood donations within three months of the initial screening visit, and the prescription of medication other than birth control. This study was approved by the Department of Kinesiology and the Texas Woman’s University Internal Review Board, and all procedures were in accordance with The World Medical Association’s Declaration of Helsinki of 1975 as revised in 1983. This study was registered at clinicaltrials.gov (NCT02634164).

### 2.2. Anthropometric, Body Composition, and Dietary Control

Basic anthropometric measures of height and weight were collected along with body composition via Dual-Energy X-ray Absorptiometry (DXA) (Lunar Prodigy GE Inc., Madison, WI, USA). At least 72 h prior to the first treatment, participants were told to abstain from physical activity, alcohol, and refrained from caffeine consumption 24 h prior to experimental conditions. For dietary control, nutritional 3-day self-recall mobile apps MyFitnessPal (www.myfitnesspal.com) was used to report dietary intake. Participants were asked to record everything they ate and drank for three days prior to each treatment day. Self-recall cell phone apps have shown similar variability when compared with conventional nutrient data collection methods such as 3-day dietary recall [39]. Additionally, all participants were instructed to maintain similarity in their dietary choices during those three days prior to each treatment day to avoid any possible related confounding factors. If participants were unable to schedule trials within one month (1 treatment per week), body composition was reassessed with DXA for accuracy of standardized amino acid dose.

### 2.3. Experimental Study Design

**Figure 1:**
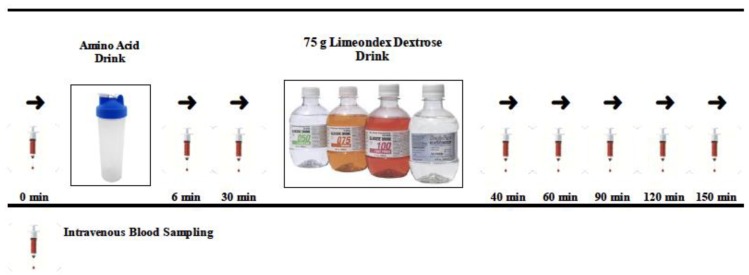
Schematic study design of intermittent intravenous sampling at baseline (0 min) to the end of the trial (150 min). Amino acid treatment (Ile, Leu, Ile + Leu, Pla) was consumed after baseline sample was taken. After 0–30 min of the amino acid only phase, dextrose (Limeondex) was consumed. Following dextrose consumption, samples were collected at 40–150 min.

The study design was a repeated measure, cross-over, singly blinded, and randomized. Following completion of the screening visit and consent, participants were randomized to complete four conditions which entailed an ingestion four treatment drinks: placebo (Pla), L-isoleucine (Ile), L-leucine (Leu), the combination of L-isoleucine and L-leucine drink (Ile + Leu). Participants were randomized in each condition in a simplistic manner where research team was blinded and selected an amino acid treatment that was color-coded prior to trial. All amino acid treatments were mixed in 150 mL of water and consumed 30 min prior to a 75 g oral glucose tolerance test (dextrose; *Limeondex*; Figure 1). All subjects arrived after a 12 h overnight fast, on the morning (0730–0800 h) of the designated treatment day. An intravenous catheter was inserted either into a posterior forearm vein or antecubital vein and blood samples were taken prior to and intermittently pre- and post-oral glucose tolerance testing (OGTT) to assess GLP-1_Active_, and GIP_Total_, glucose, insulin, C-peptide, glucagon. The analyte GLP-1_Active_ was selected as a plasma analyte of interest to minimize potential non-specific interference or cross-reactivity that has been suggested in previous literature that may lead to artificially high plasma GLP-1_Total_ values [40]. Females admitted into the study were limited to testing during the follicular phase (days 1–10) to control for any effect of the menstrual cycle and birth control on glycemia to reduce variable glycemia differences between sexes [41,42,43].

### 2.4. Amino Acids and Placebo Control Treatments

The amino acid treatments consisted of a powdered form of either Leu, Ile, and the combination of Leu + Ile (1:1 g), and a placebo. The powdered treatment formulas were fabricated by Dymatize^®^ (Dallas, TX, USA). Each dose of amino acid was appropriately weighed with an analytical scale and standardized to 0.3 g·kg^−1^ LBM bodyweight based on the efficacious dose found on glycemia in previous literature [20,35]. The amino acid treatment was consumed with deionized (DI) water (150 mL). An additional amount of DI water (50 mL) was added to the drinking utensil to rinse any leftover residue left in the drinking utensil to ensure full consumption of the dose. The placebo consisted of a powdered form of 3.54 g of inert, lecithin, sweeteners stevia and acesulfame potassium in the same composition and ratio found in the amino acid treatments. Lecithin was used to improve the dissolving of hydrophobic amino acids, Leu and Ile in the DI solution. Additionally, the sweeteners stevia and acesulfame potassium were used to increase the palatability of the drink while minimizing any possible confounding glycemic response.

### 2.5. Sampling Protocol, Storage, and Biochemistry Analysis

All blood samples were drawn without stasis from an antecubital vein (or dorsal forearm vein if present) of the right or left arm using a 20 or 22-guage indwelling Teflon catheter using a standard OGTT test with prior amino acid ingestion, that is administered for 2 h post-glucose load and is used to screen for diabetes and impaired glucose tolerance [44]. All of the samples were collected in the morning (0800–1130 h). Samples were taken intermittently at 0, 6, 30, 40, 60, 90, 120, and 150 min. The cannula was kept patent with a sterile solution of 0.9% saline. During sampling, the first mL was discarded to minimize sample dilution from saline administration. Additionally, a warming pad was wrapped around the sampling catheter for arterialization of the venous samples similar to the protocol used in previous studies [45,46,47]. Blood samples were drawn into BD Vacutainer K2 EDTA blood collection tubes and placed and chilled prior to centrifugation. In accordance with Millipore manufacturer dose recommendations, serine protease inhibitor Pefabloc^®^ SC (*11429876001*), Sigma-Aldrich Protease Cocktail Inhibitor (*P2714-1BTL*), and Sigma-Aldrich Dipeptidyl Peptidase IV (DPP-IV; *D4943 Sigma*) were prepared and added to vacutainers prior to collection of blood samples. Samples were then inverted 2–4 times, placed in a container of ice, and subsequently centrifuged at 3000 rpm at 4 °C for 10 min. Plasma samples were then transferred into storage microcentrifuge tubes and frozen at −80 °C until later analyte analysis.

The YSI 2900D Bioanalyzer (YSI Life Science, Yellow Springs, OH, USA) using enzyme electrode technology was used to determine the concentration of glucose (mmol/L) in plasma, according to the manufacturer’s maximum accuracy limits. Per manufacturer recommendations, linearity curves were generated for glucose using a calibration solution that was maintained under the manufactured calibration range max error (±5%). A volume of 25 µL of plasma was aspirated per sampling. Samples were run in duplicate with a coefficient of variation (CV%) of 19.74% ± 1.3%.

Plasma hormone concentrations were analyzed using Luminex MAGPIX platform (Luminex Corp., Austin, TX, USA) system multiplex technology following manufacturers recommendations for calibration, maintenance, and following similar to a previously used protocol [48]. For plasma hormone analysis, 25 μL of the sample was analyzed in duplicate to determine the concentration of C-peptide, GIP_Total_, GLP-1_Active_, glucagon, and insulin. This was done using a Luminex Human Metabolic Hormone multiplex assay (HMHEMAG-34K, EMD Millipore, Billerica, MA, USA). Manufacturer-supplied quality controls were included to measure assay variation and standards were set to a suggested 1:3 dilution. A minimum of ~100–400 beads (a minimum manufacture requirement of ≥32 beads) was collected for each analyte. The Magpix system was calibrated and verified before each sample analysis. The data was assessed and quantified using Milliplex Analyst Software 5.1 (EMD Millipore, Billerica, MA, USA). Millipore manufactured and supplied both standards and controls to monitor CV. Intra-assay CV was found to be <10% (3.09% ± 0.02%) for all samples run, minimum detectable concentration for each analyte was within the quality control range, and within the reported manufacturer limits: insulin 14.97 pmol/L; C-peptide 0.031 nmol/L; GIP 0.12 pmol/L; GLP-1 0.36 pmol/L.

### 2.6. Statistical Analysis

A priori power calculation was conducted (G*Power software version 3.1.9.2; Uiversität Kiel, Kiel, Germany) to estimate an appropriate sample size for the effect of leucine on glycemia. We calculated a sample size of 6 needed for a 13.6% reduction in blood glucose to a leucine ingestion and glucose load. To prepare for a possible 20% attrition rate, a total of 12 participants were recruited [30]. The statistical power (1-β error probability) was set at 0.8, α error probability at *p* = 0.05. The primary statistical analysis was a two-way repeated measure ANOVA (time × treatment) design. The effects of the treatments (Leu, Ile, Leu + Ile, Pla) on the primary dependent variables are GIP_Total_ and GLP-1_Active,_ insulin and glucose. Upon identification of a significant main effect from the RMANOVA, paired differences were evaluated from multiple comparisons when appropriate, using Tukey’s post-hoc analysis. The statistical analysis, Pearson’s product correlation coefficient, was used to assess associations between glucose, insulin, C-peptide, GIP_Total_, GLP-1_Active_, and the amino acid treatments provided. All data was expressed in mean ± SEM. Incremental change from baseline (∆) and incremental area under the curve (iAUC) for plasma glucose and hormone responses were assessed by GraphPad PRISM software (version 7.0a; GraphPad Software Inc., San Diego, CA, USA), which computes the area under the curve using the trapezoidal rule. The area equation [ΔX*(Y1*+Y*2*)/*2] formula is used repeatedly for each adjacent pair of points defining the curve for each of the test drinks ingested by each participant and analyzed in RMANOVA fashion. Incremental change from baseline data was expressed as 0–30 min (*Amino acid only phase*) and 0–150 min (*Full trial*) to compare plasma glucose, insulin, C-peptide, glucagon, GIP_Total_, and GLP-1_Active_ and calculated for each participant (treatment × time) using GraphPad. All iAUC below the baseline were excluded from the calculations [49]. Due to any potential discrepancies between analyses, we proclaimed that a true treatment effect would be confirmed if a significant difference was found in both incremental change from baseline and supported by iAUC analyses. If an assay had a missing data point, GraphPad’s program of “*interpolating from a linear standard curve*” was used to estimate the concentration value. The effect size configured by GraphPad used the standard omega squared (ω^2^) index, which estimated the proportion of variance in the dependent variable that can be explained by the independent variable. The effect sizes were determined to be: weak = 0.01, medium = 0.06, strong = 0.14 [50,51,52].

## 3. Results

### 3.1. Treatment Dose

Each participant ingested either Ile + Leu (1:1 g), Ile, Leu, and Pla treatment. The average amino acid dose was 14.68 ± 0.54 g, with a range of 11.79 to 17.61 g for each of the three amino acid treatments. The inert placebo dose was always 3.54 g for each participant.

### 3.2. MyFitnessPal 3-day Dietary Recall

Data collection and one-way ANOVA analysis of 3-day dietary intake (n = 10) showed no difference between treatments in daily macronutrient intake for carbohydrates (191.1 ± 19.7 g·day^−1^; *p* = 0.30), fat (64.5 ± 9.6 g·day^−1^; *p* = 0.84), protein (84.4 ± 24.9 g·day^−1^; *p* = 0.37), and total Kcal intake (1913 ± 361 Kcal·day^−1^; *p* = 0.42).

### 3.3. Participant Compliance and Data Integrity

Of the 14 participants were initially recruited, 12 completed. The n = 2 chose not to participate due to personal reasons. During analysis, data was omitted from a 3-day dietary recall due to participant compliancy (n = 10). Multiplexing data for plasma analysis of insulin and C-peptide (n = 11), glucagon (n = 10), GLP-1_Active_ (n = 11), and GIP_Total_ (n = 11) were omitted due to compromised data integrity from equipment failure. Following Luminex Magpix recommendations for calibration and maintenance, the aspiration probe became “clogged” during a multiplexing run, which may have compromised specific analytes and time points due to differing volume aspiration of sample timepoints or carryover. This phenomenon has been observed in this technology in previous literature by Rosenberg-Hasson et al. (2015) that observed clogging of the Luminex instrument, due to bead aggregation, which seems to be more problematic in plasma samples [53]. Therefore, due to possible compromise of these measures, they were omitted and Graphpad’s linear estimation program was used where feasible. Due to this event, and with success, we excessively added numerous timely wash steps in the protocol and sonication of the aspiration probe beyond factory recommendations to minimize similar future events.

### 3.4. Plasma Analysis

#### 3.4.1. Glucose

**Figure 2:**
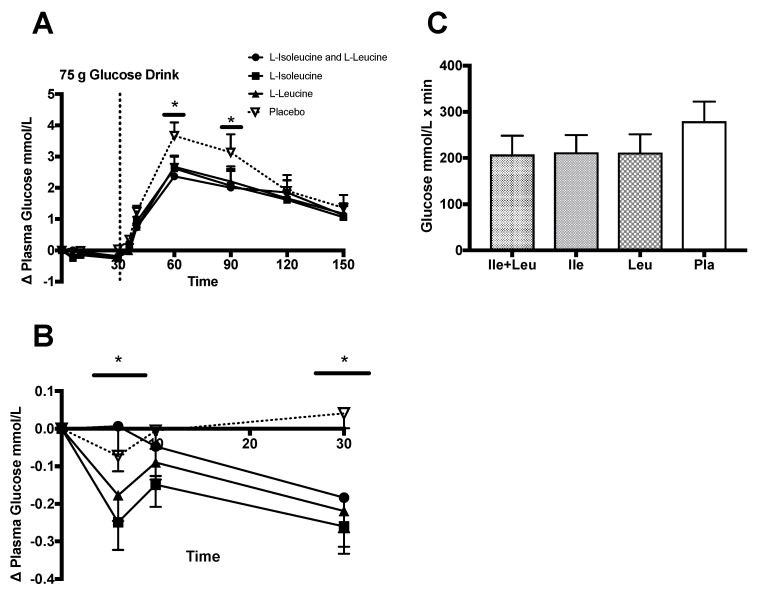
Plasma glucose (mmol/L) (mean ± SEM) comparing Ile + Leu, Ile, Leu, and Pla. (**A**; 0–150 min) main treatment effect (*p* = 0.005), (*) simple time effect differences at 60 min Ile + Leu vs. Pla (*p* = 0.008) and at 90 min Ile + Leu (*p* = 0.02) and Ile (*p* = 0.04) vs. Pla. (**B**; 0–30 min) main treatment effect (*p* = 0.01); time × treatment interaction (*p* = 0.001). (*) Post hoc Ile vs. Pla (*p* = 0.01). Simple time differences observed between Ile + Leu vs. Ile (*p* ≤ 0.0001); Ile + Leu vs. Leu (*p* = 0.007); and Ile vs. Pla (*p* = 0.01) at 6 min. At 30 min, Ile + Leu vs. Pla (*p* = 0.0007), Ile, and Leu vs. Pla (*p* ≤ 0.0001). (**C**; iAUC) No differences were found for iAUC (*p* = 0.11).

Analysis of the incremental change (∆) of plasma glucose concentration (n = 12; 0–150 min), showed no interaction between time × treatment (*p* = 0.97; ω^2^ = 0.001). There was an effect of time (*p* ≤ 0.0001; ω^2^ = 0.53) and treatment (*p* = 0.005; ω^2^ = 0.001) found on glucose responses. Further Turkey post hoc analyses revealed differences between Ile + Leu (*p* = 0.02; 95% CI −0.70 to −0.03), Ile (*p* = 0.01; 95% CI −0.71 to −0.05), and Leu (*p* = 0.02; 95% CI −0.70 to −0.03) compared to the Pla treatment and simple time effect differences at 60 min Ile + Leu vs. Pla (*p* = 0.008; 95% CI −2.3 to −0.25) and at 90 min Ile + Leu (*p* = 0.02; 95% CI −2.1 to −0.07) and Ile (*p* = 0.04; 95% CI −2.1 to −0.01) vs. Pla (Figure 2A).

The incremental (∆) change from baseline of plasma glucose concentration during the amino acid phase (n = 12; 0–30 min) prior to the glucose drink showed a significant interaction between time × treatment (*p* = 0.0013; ω^2^ = 0.06), an effect time (*p* = 0.01; ω^2^ = 0.07) and treatment (*p* = 0.01; ω^2^ = 0.07) (Figure 2B). Turkey post hoc analyses showed that Ile reduced plasma glucose (~2.5 mmol/L) more so than the Pla treatment (*p* = 0.01; 95% CI −0.28 to −0.024). Additionally, a difference was observed at 6-min post-amino acid ingestion in the Ile group compared to Ile + Leu (*p ≤* 0.0001; 95% CI 0.10 to 0.40) and Pla (*p* = 0.01; 95% CI −0.32 to −0.03). Leu showed a difference in plasma glucose concentration compared to Ile + Leu (*p* = 0.007; 95% CI 0.03 to 0.33). No significant difference was found between Ile and Leu treatments. At 30-min post-amino acid phase, all three amino acid groups had a lower glucose concentration when compared to Pla (*p* ≤ 0.0001; 95% CI Ile + Leu −0.37 to −0.07, Ile −0.44 to −0.15, Leu −0.40 to −0.11). Further iAUC analysis failed to show a concentration difference between treatments (*p* = 0.11; Figure 2C).

#### 3.4.2. Insulin and C-peptide

**Figure 3:**
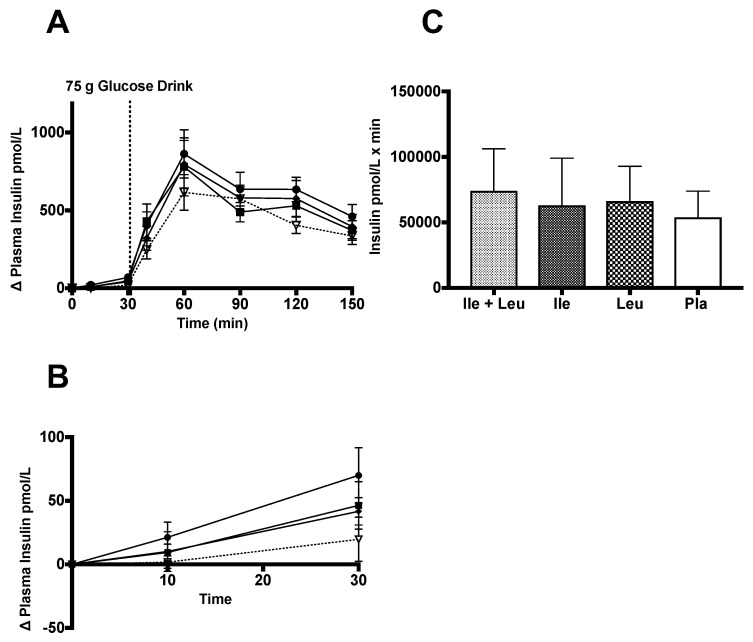
Plasma insulin (pmol/L) (mean ± SEM) comparing Ile + Leu, Ile, Leu, and Pla. (**A**; 0–150 min) no main treatment effect was found (*p* = 0.053). (**B**; 0–30 min) no main treatment effect was found. (**C**; iAUC) No other differences were found (*p* = 0.13).

The incremental (∆) plasma insulin analysis (n = 11; 0–150 min) showed no interaction of time × treatment (*p* = 0.99; ω^2^ = 0.01) or a main effect of treatment (*p* = 0.053; ω^2^ = 0.001). There was an effect of time (*p* ≤ 0.0001; ω^2^ = 0.51) (Figure 3A).

The incremental (∆) plasma insulin change during the amino acid phase (n = 11; 0–30 min) failed to show an interaction between time *×* treatment (*p* = 0.59; ω^2^ = 0.02) or a main effect of treatment (*p* = 0.13; ω^2^ = 0.003). There was an effect of time found on insulin concentration between treatments (*p* ≤ 0.0001; ω^2^ = 0.18) (Figure 3B). The iAUC analysis of insulin concentration showed no significant differences (*p* = 0.13; Figure 3C).

**Figure 4:**
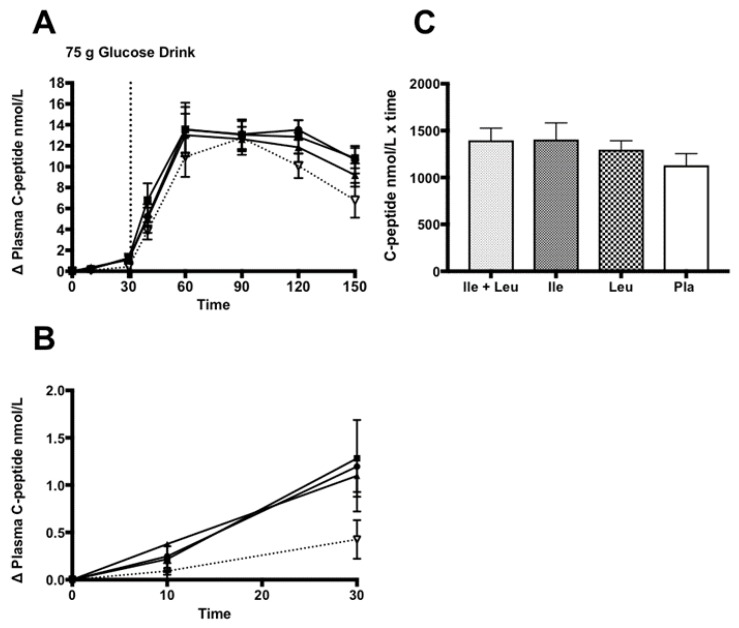
Plasma C-peptide (nmol/L) (mean ± SEM) comparing Ile + Leu, Ile, Leu, and Pla. (**A**; 0–150 min) main effect treatment (*p* = 0.04); Ile vs. Pla (*p* = 0.04). (**B**; 0–30 min) no main effect of treatment found. (**C**; iAUC) No differences were found (*p* = 0.11).

The incremental (∆) change in plasma C-peptide concentration (n = 11; 0–150 min) showed no interaction between time × treatment (*p* = 0.21; ω^2^ = 0.008). There was an effect of time (*p* ≤ 0.0001; ω^2^ = 0.65) and a treatment effect found (*p* = 0.04; ω^2^ = 0.001). Turkey’s post hoc analysis revealed that Ile had greater effect (*p* = 0.04; 95% CI 0.01 to 3.3) on C-peptide concentration than the Pla treatment (Figure 4A).

The analysis of the incremental (∆) change of plasma C-peptide concentration during the amino acid phase (n = 11; 0–30 min) also failed to show an interaction between time × treatment (*p* = 0.09; ω^2^ = 0.03), or a treatment (*p* = 0.25; ω^2^ = 0.02). An effect of time (*p* ≤ 0.0001; ω^2^ = 0.25) was found (Figure 4B). Lastly, iAUC analysis showed no differences (*p* = 0.11) between treatment groups (Figure 4C).

#### 3.4.3. Glucagon

**Figure 5:**
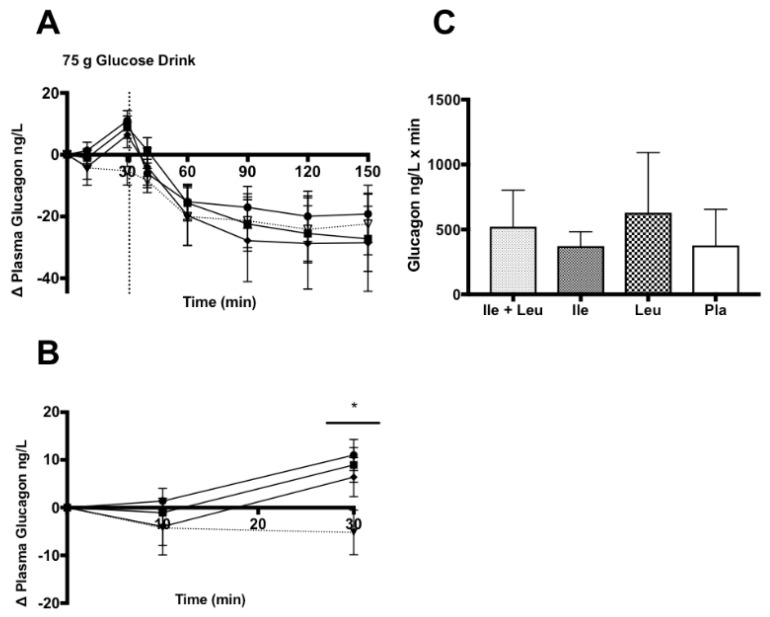
Plasma glucagon (ng/L) (mean ± SEM) comparing Ile + Leu, Ile, Leu, and Pla. (**A**; 0–150 min) no differences found. (**B**; 0–30 min) no main effect of treatment found (*p* = 0.12), time × treatment interaction (*p* = 0.04). (*) Simple time differences at 30 min Ile + Leu vs. Pla (*p* = 0.002), Ile vs. Pla (*p* = 0.001), and Leu vs. Pla (*p* = 0.01). (**C**; iAUC) No differences were found (*p* = 0.48).

The analysis of the incremental (∆) change of plasma glucagon concentration (n = 10; 0–150 min) revealed no time × treatment interaction (*p* ≥ 0.99; ω^2^ = 0.001), nor a treatment effect (*p* = 0.46; ω^2^ = 0.007). There was an effect of time (*p* ≤ 0.0001; ω^2^ = 0.19) (Figure 5A).

The incremental (∆) change analysis of plasma glucagon concentration during the amino acid phase (n = 10; 0–30 min) showed a time × treatment interaction (*p* = 0.04; ω^2^ = 0.06), an effect of time (*p* = 0.01; ω^2^ = 0.07). However, no treatment effect was found (*p* = 0.12; ω^2^ = 0.06). Simple time differences showed a greater glucagon concentration at 30 min for Ile + Leu (*p* = 0.0002; 95% CI 6.68 to 25.77), Ile (*p* = 0.001; 95% CI 4.61 to 23.68), and Leu (*p* = 0.01; 95% CI 2.04 to 21.11) when compared to Pla (Figure 5B). Further iAUC analysis showed no differences (*p* = 0.48) in glucagon concentration between treatments (Figure 5C).

#### 3.4.4. GLP-1

**Figure 6:**
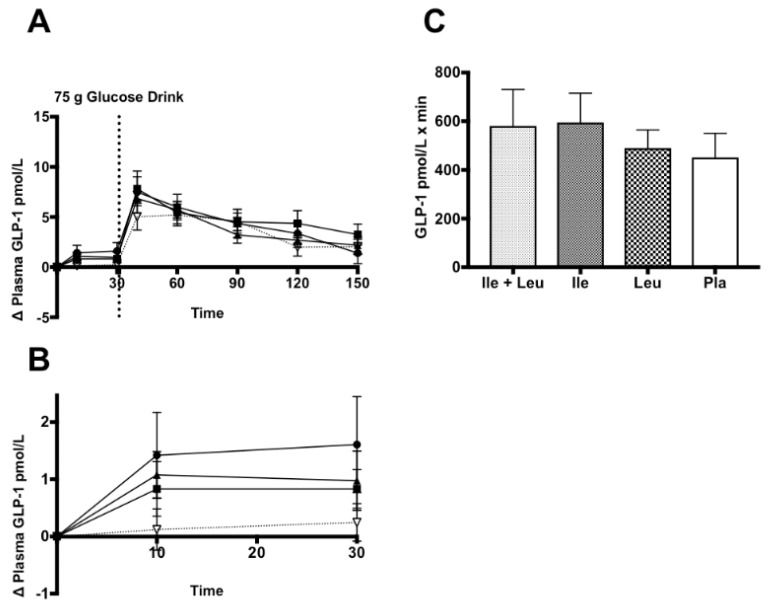
Plasma GLP-1_Active_ (pmol/L) (mean ± SEM) comparing Ile + Leu, Ile, Leu, and Pla. (**A**; 0–150 min) No differences found. (**B**; 0–30 min) No differences found. (**C**; iAUC) No differences were found (*p* = 0.31).

Incremental (∆) change in plasma GLP-1_Active_ concentration (n = 11; 0–150 min) showed no time × treatment interaction (*p* = 0.17; ω^2^ = 0.01) and no treatment effect (*p* = 0.31; ω^2^ = 0.009). An effect of time was found (*p* ≤ 0.0001; ω^2^ = 0.36) (Figure 6A).

The incremental (∆) change in plasma GLP-1_Active_ concentration during the amino acid phase (n = 11; 0–30 min) showed no time × treatment interaction (*p* = 0.45; ω^2^ = 0.02) and no main effect of treatment (*p* = 0.19; ω^2^ = 0.04). An effect of time was observed (*p* = 0.01; ω^2^ = 0.07). (Figure 6B). The iAUC analysis showed no differences (*p* = 0.31) between treatments (Figure 6C).

#### 3.4.5. GIP

**Figure 7:**
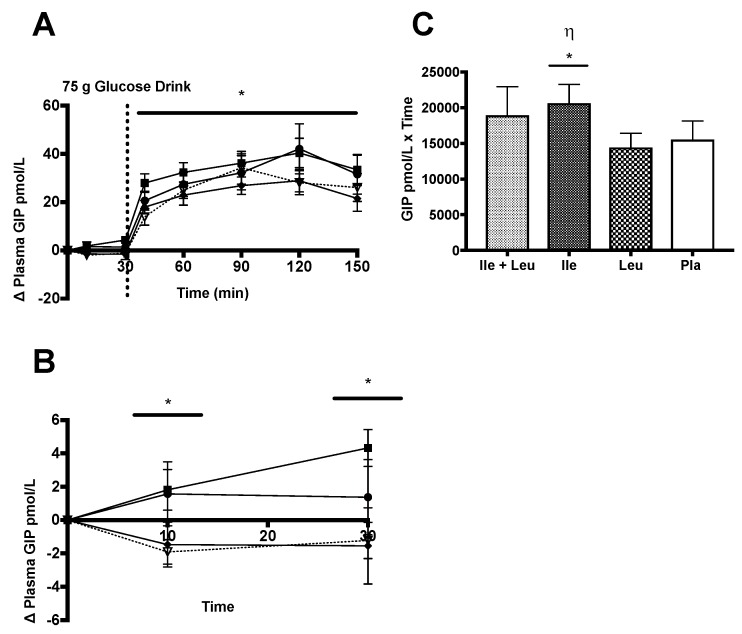
Plasma GIP_Total_ (pmol/L) (mean ± SEM) comparing Ile + Leu, Ile, Leu, and Pla. (**A**; 0–150 min) an interaction between time × treatment (*p* = 0.007), main effect of treatment (*p* = 0.01) and post hoc Ile vs. Leu (*p* = 0.02). (*) Simple time effect differences at 40 min Ile vs. Leu (*p* = 0.004) and Ile vs. Pla (*p* = 0.0001); 60 min Ile vs. Leu (*p* = 0.009); 90 min Ile vs. Leu (*p* = 0.009); 120 min Ile + Leu vs. Leu (*p* ≤ 0.0001) and Pla (*p* ≤ 0.0001), Ile vs. Leu (*p* = 0.0008) and Pla (*p* = 0.0003); 150 min Ile + Leu vs. Leu (*p* = 0.004) and Ile vs. Leu (*p* = 0.0005). (**B**; 0–30 min) A interaction between time and treatment (*p* = 0.03) and main treatment effect was found Ile vs. Leu (*p* = 0.04) and Pla (*p* = 0.04). (*) Simple effect differences were found at 10 min Ile + Leu vs. Pla (*p* = 0.04) and Ile vs. Pla (*p* = 0.02); at 30 min Ile vs. Leu (*p* = 0.0002) and Ile vs. Pla (*p* = 0.0004). (**C**; iAUC) Plasma GIP_Total_ (pmol/L × time): (η; *) main treatment effect (*p* = 0.04); Ile vs. Leu (*p* = 0.03) and Ile vs. Pla (*p* = 0.008).

Analysis of the incremental (∆) change of plasma GIP_Total_ concentration (n = 11; 0–150 min) showed a time × treatment interaction (*p* = 0.007; ω^2^ = 0.001), a main effect treatment *p* = 0.01; ω^2^ = 0.02), and an effect of time (*p* ≤ 0.0001; ω^2^ = 0.49). Further Turkey post hoc analysis revealed that Ile had a greater concentration of GIP_Total_ compared to Leu (*p* = 0.02; 95% CI 0.85 to 14.46). Simple time differences of concentration of GIP_Total_ were found at 40 min (Ile > Leu; *p* = 0.004; 95% CI 2.31 to 17.65, and Pla *p* ≤ 0.0001; 95% CI 6.33 to 21.68), 60 min (Ile > Leu; *p* = 0.009; 95% CI 1.719 to 17.06), 90 min (Ile > Leu; *p* = 0.009; 95% CI 1.67 to 17.02), 120 min (Ile + Leu > Leu; *p* ≤ 0.0001; 95% CI 5.53 to 20.88, Ile + Leu > Pla; *p* ≤ 0.0001; 95% CI 6.36 to 21.71, Ile > Leu; *p* = 0.0008; 95% CI 3.81 to 19.15, and Ile > Pla; *p* = 0.0003; 95% CI 4.63 to 19.98), and 150 min (Ile + Leu > Leu; *p* = 0.004; 95% CI 2.36 to 17.71, and Ile > Leu; *p* = 0.0005; 95% CI 4.25 to 19.6) (Figure 7A).

Analysis of the incremental (∆) change of plasma GIP_Total_ during the amino acid phase (n = 11; 0–30 min), showed a time × treatment interaction (*p* = 0.03; ω^2^ = 0.05) and a main effect of treatment (*p* = 0.02; ω^2^ = 0.08). No effect of time was found (*p* = 0.72; ω^2^ = 0.006). Turkey’s post hoc analysis showed a higher concentration of GIP_Total_ in the Ile treatment compared to Leu (*p* = 0.04; 95% CI 0.01 to 6.09) and Pla (*p* = 0.04; 95% CI 0.05 to 6.13) (Figure 7B). Simple time differences showed Ile + Leu > Pla (*p* = 0.04; 95% CI 0.03 to 6.92), Ile > Pla (*p* = 0.02; 0.28 to 7.17) at 10 min and Ile > Leu (*p* = 0.002; 95% CI 2.42 to 9.31), Ile > Pla (*p* = 0.0004; 95% CI 2.1 to 9.31) at 30 min. Furthermore, iAUC analysis resulted in a difference (*p* = 0.04) between treatments, where Ile had a greater GIP_Total_ concentration than Leu (*p* = 0.03) and Pla (*p* = 0.008) (Figure 7C).

#### 3.4.6. GIP Correlations

**Figure 8:**
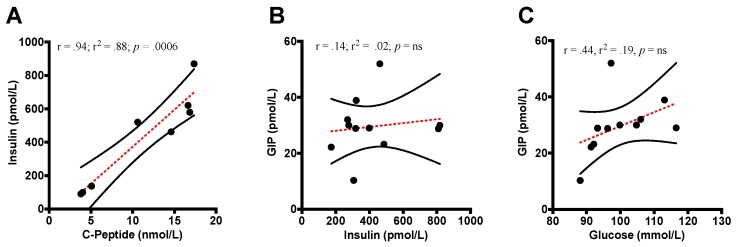
Correlations between insulin, C-peptide, glucose, and GIP (mean ± SEM) (**A**) plasma insulin (pmol/L) and C-Peptide (nmol/L) (r= 0.94; r^2^ = 0.88; *p* = 0.0006); (**B**) plasma GIP (pmol/L) and insulin (pmol/L) (*r* = 0.14; *r^2^* = 0.02; *p* = ns); (**C**) plasma GIP (pmol/L) and glucose (mmol/L) (*r* = 0.44; *r^2^* = 0.19; *p* = ns).

There was no association found between GIP concentration during the Ile treatment in plasma GIP and glucose concentrations (Figure 8C: *r* = 0.44, *r^2^* = 0.19, *p* = ns) and between insulin and GIP (Figure 8B: *r* = 0.14; *r^2^* = 0.02; *p* = ns). A correlation was found between insulin and C-peptide (Figure 8A: *r* = 0.94, *r^2^* = 0.88, *p* = 0.0006).

## 4. Discussion

The purpose of this study was to determine the effects of large, orally ingested dose of L-Leucine (Leu) and L-Isoleucine (Ile) individually or equally combined, on the plasma concentration of incretins glucagon-like peptide-1 (GLP-1) and glucose-dependent insulinotropic peptide (GIP), the pancreatic hormones insulin and glucagon, and their overall impact on glycemia prior to and after a standard CHO drink. The main findings of this study showed that the pre-ingestion of Ile and Leu, in combination and individually, have differing incretin effects, had no notable effect on insulin concentrations, and likewise minimal impact on glucose concentration. However, there was an amino acid-induced reduction in glucose during the peak time points at 60–90 min post-CHO consumption. Additionally, our results show that Ile seems to stimulate GIP release more so than Leu, or in combination with Leu. However, an increase in GIP concentration did not seem translate to a greater response in insulin. Therefore, based on our outcomes, it appears that pre-ingested Leu and Ile minimize the magnitude of the peak time points of post-CHO drink hyperglycemia, Ile-stimulated GIP release does not seem to promote greater insulin release and overall no meaningful impact on glucose concentrations during a standard OGTT.

Due to differences in study designs, the models used for amino acid and glucose administration (i.e., pre-ingestion vs. co-ingestion, oral ingestion vs. intraduodenal vs. intragastric), the dose of amino acid and glucose or drink used, and use of either individual amino acids or a mixture of amino acids, it is difficult to fairly compare our results to prior research studies. To facilitate, simplify, and more fairly compare to previous literature, we separated our results discussion into 0–30 min (*Amino acid only phase*) and the full 0–150 min (*Full trial*) that included dextrose ingestion for each outcome assessed. Additionally, this delineation better compares the glycemic and hormonal responses from amino acid ingestion and how it compares to and impacts the subsequent ingestion of CHO.

### 4.1. Glucose

Our observation of the plasma glucose response was slightly different than previous research observing the oral ingestion of amino acids Leu and Ile [30,35]. During the 30 min amino acid phase, after the ingestion of Ile + Leu, Ile, and Leu, we did see a significant difference, yet it could be debated how physiologically meaningful a concentration reduction of 0.2 mmol/L of blood glucose is. Nuttall et al. (2008) co-administered 25 g of glucose with ~7 g of Ile and observed an 8% reduction in blood glucose (4.66 to 4.28 mmol/L) [35]. Our Ile results showed a reduction of plasma glucose to a lesser magnitude −0.26 mmol/L. These differences may be partially explained by the total time observed after Ile ingestion, dose, and the sample of participants. We did not find any significant changes in plasma glucose by Leu, and the combination of Ile + Leu during the 0–30 min phase of the trial when compared to Pla or Ile. Kalogeropoulou et al. (2008) reported no significant or meaningful glucose changes with Leu when ingested alone compared to their control treatment of water [30].

Observing the full trial (0–150 min), glucose concentration rose from a baseline (4.97 mmol/L) value and peaked at 60 min at ~7.5 mmol/L. Glucose then fell to ~5.9 mmol/L at the end of treatment (150 min). Compared to Pla, all amino acid treatments compared to placebo reduced plasma glucose at the peak time points of 60 and 90 min by ~1.2 mmol/L. Interestingly, glucose concentrations in all treatment groups remained above baseline (18%) until the end of the trial. Interestingly, the Pla group had a similar response as the amino acid treatment groups, in that it did not return to baseline after 150 min. Overall, this suggests that the sample population that participated in this study may have had some level of glucose intolerance and possibly a reduction in insulin sensitivity. Hamburg et al. (2009) showed that 5 d of physical inactivity in a healthy group of males and females influenced higher glucose and insulin (67%) concentration during an OGTT test [54].

Similar to Ullrich et al. (2016), we showed that Leu and Ile independently and in combination reduced the blood glucose when administered alone and reduced the hyperglycemic peak time points at 60 and 90-min ~1.2 mmol/L after the consumption of the CHO drink. They reported a Leu and Ile induced 1.1 mmol/L reduction in glycemia post-macronutrient drink consumption. Steinert et al. (2015) reported a slight decrease in blood glucose from the higher dose intradoudenal infusion rate. In concert with both Steinert et al. (2015) and Ullrich et al. (2016), it appears as though Leu and Ile seem to promote a slight blood glucose reduction and minimize the magnitude of hyperglycemia if ingested prior to a macronutrient drink [36,37].

The glucose area (iAUC) analysis showed no differences between treatments. It appears that the amino acid treatments at the chosen doses and ingestion protocol overall were not impactful enough to elicit an area reduction in glucose compared to Pla or the study design may have lacked the appropriate number of participants even after priori power estimation to show a difference. However, the effect size found showed the treatment effect to be weak. Kalogeropoulou et al.’s (2008) analysis of the co-ingestion of Leu and glucose iAUC showed a 50% reduction in the area of glucose in comparison to our data where we showed no iAUC differences between treatments. Taking into consideration the differences in protocols, doses, analysis, inter- and intrasubject variability from previous research study designs; it seems as though prior models administering Ile or Leu with a 25 g dose of glucose may elicit a stronger effect on reducing the blood glucose than does the ingestion of a larger dose of amino acids prior to a larger glucose drink [30,35].

### 4.2. Insulin and C-peptide

During the 30-min amino after acid only phase, we showed no differences between treatments. This outcome was similar to Nuttall et al. (2008) who showed no difference in the insulin response from Ile compared to their control treatment of water ingestion. Interestingly, the mean dose if Ile for their study was ~7.4 g compared to our study design with a mean dose of 14.6 g. The larger dose of Ile did not elicit a meaningful insulin response. During the 30-min phase of Leu ingestion, we did not find an effect of treatment compared to Pla on insulin response. This finding was in contrast to Kalogeropoulou et al.’s (2008) that observed a significant increase in insulin concentration with the ingestion of Leu compared to their control treatment of water [30,35]. Our results were similar and in contrast to Ullrich et al. (2016) in that we did not find an insulin response to Leu nor Ile. They found a slight rise in insulin during the intragastric administration of 5, 10 g of Leu, yet not with Ile. Steinert et al. (2015) reported a slight increase in insulin during intradoudenal administration of Leu [36,37].

Our analysis of the full trial showed that a pre-ingestion of Ile and Leu induced a higher peak insulin concentration response than what Nuttall et al. (2008) and Kalogeropoulou et al. (2008) showed with the co-ingestion of 25 g glucose with ~7.4 g of either Ile or Leu, respectively. The contrasting outcomes may be explained by the drink selected (*Ensure* vs. *Limeondex*), dose of CHO (25 vs. 75 g), amino acid dose (~7.4 vs. 5–10 vs. ~14.6 g), the timing of amino acid ingestion, and inter- and intra-variability in the sample of participants.

The insulin response from the combination of Ile + Leu, was similar to that of what Nilsson et al. (2007) concluded. With notable methodological differences between studies, their participants showed a peak increase in insulin concentration of ~400 pmol/L, 30 min after co-ingestion of 25 g of glucose and 4.4 g of BCAA’s. In contrast, we did not find a statistical difference between amino acid treatments on insulin responses. Our data showed no differences between any treatments concurrent with a weak effect size. Our iAUC analysis did not find a difference in insulin concentration with the pre-ingestion of these amino acids. Therefore, it appears that Ile and Leu in a larger dose, individually ingested, or in combination, prior to a 75 g CHO drink, does not seem to influence insulin concentration differently than Pla. However, it could be argued, similar to the glucose outcomes, due to the sample number recruited, this may have affected the power of the statistical analysis of the study. However, with a weaker effect size observed, it appears that these amino acids, and the prescribed dose and protocol, do not stimulate a greater insulin response. Furthermore, to investigate any impact on insulin sensitivity by Leu and Ile, we assessed insulin sensitivity (OGIS) using a prior model [55] and found no difference between treatments (data not shown). Based on our analysis, Ile and Leu have no impact on insulin concentration and sensitivity.

The analysis of C-peptide during the 30 min Ile + Leu, Ile, and Leu ingestion, C-peptide concentration minimally increased ~1.3 nmol/L. No difference was found between amino acid treatments, in that they all influenced a very small increase C-peptide similarly in the amino acid only phase. It appears that Ile and Leu may stimulate C-peptide concentration. However, it could be speculated that such a small increase in C-peptide may not be physiologically meaningful and requires more work to observe any thresholds. During the full trial, no significant differences were found. This was further supported by iAUC analysis did not show a difference in C-peptide concentration between any treatment. Our results were unlike Lindgren et al. (2015), that found an increase concentration (600 pmol/L) at ~30 min and a two-fold rise in C-peptide AUC concentration due to the ingestion of amino acid mixture [21].

It appears as though Ile and Leu, independently or in conjunction, prior to CHO ingestion, have any impact on C-peptide concentration.

### 4.3. Glucagon

The glucagon concentration response to Ile + Leu, Ile, Leu during the 30 min amino acid only phase resulted in a small increase from baseline of 54 to 61.9 ng/L and similar to Nuttall et al. (2008) and Steinert et al. (2015). Nuttall et al. (2008) showed a slight increase in glucagon from Ile ingestion 0–30 min phase ~53 to 60 ng/L. Ullrich et al. (2016) showed an increase ~65 to 80 pg/mL after Leu administration while no difference was found for Ile. Steinert et al. (2015) showed no effect on glucagon during intraduodenal infusion. Our glucagon response to Ile and Leu seemed to be somewhat similar with the observations of Kalogeropoulou et al. (2008) and Nuttall, et al. (2008), that showed to stimulate a slight rise in glucagon concentrations at 30 min post-amino acid ingestion to a differing degree. It appears as though Leu stimulated a greater glucagon concentration rise than did Ile when consumed individually. In contrast, we observed no difference between amino acid treatments at 30 min time point on glucagon concentration [30,35].

During the full trial (150 min) and after the consumption of CHO drink, no other differences were found between treatments on glucagon concentrations. Similar to other previously addressed responses, glucagon never returned to baseline values and remained depressed, which may be explained by the concurrent hyperinsulinemia that remained above baseline until the cessation of the trial.

In conjunction with previous studies, Ile and Leu independently and concurrently stimulated a slight increase in glucagon concentration. After CHO ingestion, as expected, glucagon concentration fell concurrent with the rise in insulin concentration where no differences were found between treatments. This response, as previously explained, is considered normal in a healthy, non-diseased population, where insulin is antagonistic to the secretion of glucagon [56].

### 4.4. GLP-1

To our knowledge, there are only a few studies that have used a human model and assessed the impact of individual Ile and Leu ingestion on GLP-1 concentration [36,37]. Multiple investigations have observed the effect of either whey protein, amino acid mixtures, and L-Glutamine on active and/or total incretin responses with mixed outcomes.

Our data showed no difference between treatments during the amino acid phase on GLP-1 concentration. Furthermore, our analysis of the GLP-1 during the full trial of all treatments showed no differences either. This suggests that Leu and Ile may not factor in the previously seen whey protein-induced stimulus and secretion of GLP-1 [13]. Similar to Nilsson et al. (2007), who observed no effect of 4.4 g of BCAAs and 25 g of glucose on GLP-1 concentration [57]. With distinct differences in our protocol and dose compared to Nilsson et al. (2007), it appears that the Ile and Leu have no impact on GLP-1, which suggests that either another amino acid or group of amino acids, or dipeptides found in whey protein, may stimulate GLP-1 secretion and/or DPP-IV inhibition [58,59].

Ullrich et al. (2016) similarly showed no difference 20 min after intragastric administration of Leu and Ile compared to baseline. After the ingestion of their multiple macronutrient enriched drink (*Ensure*), the GLP-1 response was not enhanced by the ingestion of Leu or Ile. They suggested that the due to the normal glucose concentration response seen in their healthy, normal population (<8 mmol/L), the incretin promoted insulinotropic would not have been likely seen [60]. Concurrent, with their suggestion, we showed a similar peak glucose concentration of 7.5 mmol/L and no difference found in GLP-1 nor insulin concentrations between trials.

In parallel, Steinert et al. (2015) that showed no impact on GLP-1 through intradoudenal administration of Leu either [36,37]. Interestingly, with differing methodologies between the studies highlighted, Leu and Ile do not seem to impact GLP-1 active or total concentration.

The lack of Ile and Leu-induced GLP-1 response may be a related to time and interaction. Roughly ~50% of BCAA’s may be directly extracted by splanchnic tissues for metabolic use while the other portion is delivered to the systemic system [61]. A factor that may influence the interaction of the ingested amino acids with L-cells of the lower intestine and colon where GLP-1 is synthesized has been posited as the “*transit theory*”. This theory was previously suggested by Lindgren et al. (2015), that stated the duration of interaction between amino acids and the L-cells may assist in explaining why GLP-1 was unaffected. Additionally, it could be postulated that the difference in ingestion protocols of CHO and the amino acids may have stimulated a different response [21,62]. However, Lindgren et al. (2015) compared intraduodenal and intravenous introduced amino acid mixtures on GIP, GLP-1, glucagon, insulin, and blood glucose. Similar to our outcomes, they did not see an effect of the amino acid mixture treatment on intact or total GLP-1 responses [21]. This further supports that amino acids do not affect GLP-1 responses, regardless of its introduction.

Interestingly, our data was found to be in contrast to previous research using a human intestinal cell culture model that showed that using a 3% concentration of Leu and Ile increased the active form of GLP-1 474% and 264%, respectively [63]. It seems as though in a cell model, Leu and Ile are able to stimulate a robust GLP-1 secretion. Furthermore, the relationships with protein ingestion and GLP-1 release becomes more convoluted with the ingestion of whey, in that other factors such as dipeptides found in whey may both inhibit the actions of DPP-IV [58], and stimulate GLP-1 secretion [59]. Lastly, Tulipano et al. 2011 showed that a peptide found in β-lactoglobulin, which is a predominant protein found in whey, moderately inhibits DPP-IV activity, or slows GLP-1 degradation [64]. In summary, there seems to be a disconnect between whole protein ingestion, amino acid ingestion, human vs. in vitro models and their influences on GLP-1 release. This suggests that whey protein-induced GLP-1 release may be facilitated by other amino acids, a mixture of amino acids, dipeptides found in whey that inhibit DPP-IV activity.

### 4.5. GIP

Additional to previous literature investigating individual amino acids on GLP-1 responses, to our knowledge, only a few studies have assessed the effect of Leu and Ile on total GIP concentration. During the 30 min amino acid only phase, post-Ile ingestion, GIP increased ~37% when compared to both Pla and Leu treatments. Furthermore, similar to the Ile treatment, the combination of Ile + Leu did not seem to have an effect on GIP. Considering that Leu had no impact on GIP concentration when ingested alone, it may be feasible to suggest that the combination treatment of Ile + Leu may have had no effect due to Leu concentration in the mixture.

In conjunction with the outcomes found during the 30 min Ile amino acid only phase, the full trial showed that Ile again influenced the GIP concentration compared to the other treatments. This outcome supports previous work by Lindgren et al. (2015) who found that amino acid ingestion did induce a GIP response. However, their protocol used a multiple amino acid mixture solution, with half the dose used in our study design. Their data suggests that other amino acids in the ingested mixture independently or in combination induced a GIP response [21]. Furthermore, Nilsson et al. (2007) showed a greater GIP response after the co-ingestion of BCAA’s with glucose more so than any other treatment. Our data parallels the outcomes found by Lindgren et al. (2015) and Nilsson et al. (2007), that amino acids do stimulate GIP release. Interestingly, our outcome was in contrast to Ullrich et al. (2016) who found that intragastric Leu administration promoted an increase in GIP compared to Ile. However, after the ingestion of their multiple macronutrient enriched drink, they did not find a difference between treatments. The differing outcomes between studies may be potentially explained by methodological differences, yet the common theme is that amino acids seem to be a factor in stimulating GIP release.

In assessing the correlative relationships during the Ile treatment, it appears that the rise in plasma GIP did not associate with the rise in glucose and insulin concentrations. This is in contrast to previous research finding strong correlation. However, the previous study used glucose alone without any amino acid ingestion [65]. Based our outcomes, we found no relationship between Ile-stimulated GIP concentration and subsequent insulin concentration. It has been widely accepted and acknowledged that glucose-driven GIP secretion does indeed stimulate β-cell receptor secretion of insulin [22,66]. However, our Ile-stimulated GIP outcome was contrary to the relationships found by Wachters-Hagedoorn et al. (2006), who noted a strong positive correlation between GIP and the rate of glucose appearance, as well as between insulin rate of appearance [67]. However, based on previous nutritional research and our outcomes, it appears as though amino acids do promote GIP release; yet the GIP-induced insulin release is primarily dependent on glucose ingestion opposed to amino acid ingestion [68].

A potential mechanism to explain the Ile-induced GIP increase in concentration may involve amino acid exchange hypothesis. This exchange would involve aliphatic L-amino acid and oligopeptide sensing sensitive G protein-coupled receptors (GPCRs) found in the GI tract. The GPCRs are more specifically named the calcium-sensing receptor (CaSR). The CaSR are associated with a variety of gut hormones such as GIP, and GLP-1, which have been strongly linked to incretin hormone secretion in vitro and in vivo, and are the principle Gαq-coupled receptor that is activated by Ca^2+^, aromatic amino acids, and oligopeptides [69]. The presumed model of incretin exocytosis is Na^2+^ and Ca^2+^ dependent action potential-induced membrane depolarization, which is accompanied by a voltage-dependent Ca^2+^ influx that occurs partially through Ca^2+^ channels (L-type cells). These cellular actions are suggested to be essential for triggering the release of GIP and GLP-1 secretory granules [70]. Furthermore, intestinal aromatic amino acid transporters (TAT1/SLC16A10) are not involved in the transport process, substantially expressed, and observed in all sections of the small intestine. The expression of TAT1 increases toward the tip of the villi and is observed in the basolateral membrane, which could contribute to a net efflux of other neutral amino acids and in turn allows for the efflux of aromatic amino acids, which may then influence the exchange for other neutral amino acids (i.e., Ile), via system L-amino acid transporters (LAT2/SLC7A8) [71]. This amino acid exchange hypothesis may assist in explaining the effect Ile has on GIP secretion. However, this hypothesis is purely speculation and more research is needed to isolate and explain these relationships.

In addition to our findings, there are numerous limitations that need to be considered. Due to the sample number of participants observed and known variation in human responses to nutrition, a larger sample may have led to more power to see difference in treatments. However, power analysis estimated ~6 participants were needed to see glycemic reduction, which further highlights the poor impact of the pre-CHO-ingested amino acid model. Another variable that was not assessed nor controlled for is the gastric emptying rate. One of the more paramount procedures to control for variability in metabolic blood concentrations is to observe and standardize gastric emptying rates. It has been suggested in normal, healthy persons, there are intersubject (37%) and intrasubject (13%–35%) variabilities in gastric emptying rates after the consumption of liquid [72]. Additionally, previous research has shown that there are indeed differences in blood glucose, insulin, incretins, and gastric motility in healthy persons with glucose consumption and this should be considered and cautioned before extrapolating our data onto other populations of interest [73]. However, with the overarching goal and focus of practicality applied in our study design, we sought to investigate what may be typically seen during oral ingestion of a large bolus of Ile, Leu, and CHO.

The GIP response that was assessed in our investigation were both the intact (active) and degraded (inactive) forms termed GIP_Total_. We may have seen a similar outcome in our GLP-1 analysis if we had selected the GIP_Active_ analyte opposed to the GIP_Total_. Per Millipore manufacturing requirements, we were unable to analyze both active and inactive forms of both incretin analytes in the same kit. For example, it may have been possible that a difference may have been seen using GLP-1_Total_ in comparison to GLP-1_Active_ and similarly, no difference seen using GIP_Active_ compared to GIP_Total_. However, in conjunction with previous studies assessing active and total GLP-1 concentrations, our data does support that amino acids do not seem to have an impact.

Lastly, another study limitation that would further support our proposed suggestions and explanations is the analysis of amino acid concentration in plasma associated with the concentration hormones and glycemia. Previous research has strengthened its conclusions with plasma concentrations of specific amino acids related to glycemic alterations.

## 5. Conclusions

Our data suggest that L-isoleucine may play a significant role in amino acid-driven GIP concentration, while L-leucine does not seem to have the same effect. In concert with previous investigations, neither L-isoleucine nor L-leucine seem to have an effect on GLP-1 concentration. This further supports that another agent in whey protein seems to be influencing GLP-1 appearance. Moreover, the pre-ingestion of a large bolus of L-isoleucine or L-leucine prior to the consumption of carbohydrates does not mitigate any notable hyperglycemic responses compared to the previous co-ingestion of carbohydrate and amino acid models. However, it may reduce peak time points normally seen after the ingestion of a carbohydrate drink. Moreover, it is unknown if the hyperglycemia and hyperinsulinemia seen at the cessation of trial is due to ingestion of treatments or sample population. Lastly, our observations show that L-isoleucine driven-GIP concentration has no relationship with the insulin concentration, which is in contrast to ingested glucose driven-GIP secretion. Therefore, the peak reduction that was found on blood glucose cannot be explained by an insulin response because the insulin area was found to be no different than the placebo control trial. Overall, it does not appear that the pre-ingested model of L-isoleucine and/or L-leucine prior to carbohydrate intake has a meaningful effect on glycemia and its related pancreatic hormones. Compared to previous literature, whey protein and amino acid mixtures co-ingested with carbohydrates, in a specific ratio, seem to promote a more favorable glycemic response.

## Figures and Tables

**Table 1:** Participant Characteristics.

Number of Participants (M/F)	12 (6/6)
Age (years)	27.3 ± 2.0
Height (cm)	167.4 ± 2.2
Weight (kg)	77.3 ± 3.7
BMI (kg/m^2^)	26.3 ± 2.1
LBM (kg)	48.6 ± 1.8
Body Fat (%)	34.1 ± 2.9
Glucose (mmol∙L^−1^)	4.97 ± 0.09
Glucose (mg∙dL^−1^)	89.5 ± 1.7
Insulin (pmol∙L^−1^)	96.8 ± 13.3
Glucagon (ng∙L^−1^)	54.1 ± 9.7
GIP_Total_ (pmol∙L^−1^)	12.3 ± 2.1
GLP-1_Active_ (pmol∙L^−1^)	1.9 ± 0.39

Values are reported in mean ± SEM.

## Data Availability

The data that support the findings of this study are available from the corresponding author upon reasonable request.
